# DREAM Is Involved in the Genesis of Inflammation-Induced Prolabour Mediators in Human Myometrial and Amnion Cells

**DOI:** 10.1155/2018/8237087

**Published:** 2018-02-22

**Authors:** Priyanka Goradia, Ratana Lim, Martha Lappas

**Affiliations:** ^1^Obstetrics, Nutrition and Endocrinology Group, Department of Obstetrics and Gynaecology, University of Melbourne, Melbourne, VIC, Australia; ^2^Mercy Perinatal Research Centre, Mercy Hospital for Women, Heidelberg, VIC, Australia

## Abstract

Preterm birth is the primary cause of perinatal morbidity and mortality worldwide. Inflammation induces a cascade of events leading to preterm birth by activating nuclear factor-*κ*B (NF-*κ*B). In nongestational tissues, downstream regulatory element antagonist modulator (DREAM) regulates NF-*κ*B activity. Our aims were to analyse DREAM expression in myometrium and fetal membranes obtained at term and preterm and to determine the effect of DREAM inhibition on prolabour mediators in primary myometrial and amnion cells. DREAM mRNA expression was significantly higher in fetal membranes obtained after spontaneous labour compared to nonlabour and in amnion from women with histological preterm chorioamnionitis when compared to amnion from women without chorioamnionitis. In primary myometrial and amnion cells, the effect of DREAM silencing by siRNA was a significant decrease in the expression of proinflammatory cytokine IL-6, the chemokines IL-8 and MCP-1, the adhesion molecule ICAM-1, MMP-9 mRNA expression and activity, and NF-*κ*B transcriptional activity when stimulated with the proinflammatory cytokine IL-1*β*, the bacterial products fsl-1 or flagellin, or the viral dsRNA analogue poly(I:C). These data suggest that, in states of heightened inflammation, DREAM mRNA expression is increased and that, in myometrial and amnion cells, DREAM regulates proinflammatory and prolabour mediators which may be mediated via NF-*κ*B.

## 1. Introduction

Preterm birth, defined as delivery prior to 37 weeks of gestation, affects approximately 15 million pregnancies annually and is the primary cause of perinatal morbidity and mortality worldwide [1]. Spontaneous preterm birth accounts for up to 70% of all cases [2]. Globally, more than one million preterm babies die each year, and those who survive have significantly higher rates of health complications, such as respiratory distress, jaundice, cerebral palsy, and cognitive impairments, compared to those born at term [3, 4]. Intensive short- and long-term care for these babies poses a significant economic challenge, while the emotional toll borne by their families, also, is immense [5]. Despite clinical interventions and extensive research, preterm birth rates continue to rise. A more thorough understanding of the mechanisms of human parturition is essential to designing new and effective strategies for the prevention and management of preterm labour.

Healthy term labour is widely acknowledged to be a physiological, inflammatory state characterised by leukocytic infiltration of the myometrium, cervix, and fetal membranes [6, 7]. The subsequent release of proinflammatory cytokines, such as interleukin- (IL-) 1*β*, facilitates the processes of parturition, with elevated levels found in myometrium, amnion, cervical tissue, and amniotic fluid in association with labour [7, 8]. IL-1*β* can amplify production of cytokines and chemokines, including IL-8 and monocyte chemoattractant protein-1 (MCP-1), and upregulate the expression of cell adhesion molecules such as intercellular adhesion molecule-1 (ICAM-1), which promotes further leukocyte recruitment [9]. They also induce cyclooxygenase-2 (COX-2) expression, resulting in uterotonic prostaglandin production [10, 11], and that of matrix metalloproteinase- (MMP-) 9, an extracellular matrix remodelling enzyme implicated in cervical ripening and membrane rupture [12]. Spontaneous preterm birth is thought to result from untimely and pathological activation of this pathway due to infection, haemorrhage, uterine distention, obesity, or stress, among others [2, 13]. These disease processes share the feature of amplified local or systemic inflammation. Of these, infection has the greatest clinical significance [14] and initiates the aforementioned cascade of inflammatory events via toll-like receptor (TLR) activation by bacterial or viral products.

The nuclear factor-kappa B (NF-*κ*B) signalling pathway is classically associated with inflammation and, in gestational tissues, is a critical regulator of prolabour mediators [15, 16]. NF-*κ*B is highly inducible by IL-1*β* and microbial products, while NF-*κ*B recognition elements are found within genes encoding IL-1*β*, IL-6, IL-8, and TNF-*α*, creating a positive feedback loop. Expression of the RelA subunit is significantly increased in myometrium and amnion in association with labour [17, 18]. Furthermore, NF-*κ*B inhibition has been shown to dampen expression of prolabour mediators in response to proinflammatory stimuli in myometrium, amnion, and placenta [19–21] and also delay time to delivery in mice [22]. Thus, controllable modulation of NF-*κ*B signalling may be of value in preventing spontaneous preterm birth.

Downstream regulatory element antagonist modulator (DREAM), also known as calsenilin and KChIP3, belongs to the neuronal calcium sensor family and has recently been shown to play a role in NF-*κ*B signalling [23–25]. It primarily exists in the cytosol, but nuclear and plasma membrane localisation have also been reported [26, 27]. Three isoforms are known to exist, with molecular weights of 29.1 kD, 26.7 kD, and 26.3 kD. Initial identification of DREAM encompassed various biological processes, including senile plaque production in Alzheimer's disease, pain sensation, membrane excitability, and synaptic plasticity [27–29]. DREAM-deficient mice consistently display attenuated responses to inflammatory pain models and have decreased levels of NF-*κ*B-transcribed proinflammatory mediators in animal models of inflammatory lung and vascular injury [24, 25, 30]. Additionally, DREAM has been shown to bind to promoters of anti-inflammatory cytokines, suppressing their transcription [31]. Only one study has investigated the role of DREAM in pregnancy tissues. DREAM mRNA expression is upregulated in placentas from women with severe early onset preeclampsia [32], another adverse pregnancy outcome associated with inflammation.

Given the central role of inflammation and NF-*κ*B signalling in the processes of human labour and delivery, it was hypothesised that (i) human labour and infection would be associated with increased DREAM expression in human myometrium and fetal membranes and (ii) DREAM silencing would be associated with decreased expression and release of prolabour mediators in the presence of proinflammatory stimuli. Thus, the aims of this study were to (i) characterise the expression of DREAM in human myometrium and fetal membranes obtained from labouring and nonlabouring women at term and preterm with and without evidence of infection and (ii) determine the effect of DREAM silencing on prolabour mediators in human primary myometrial and amnion cells. The proinflammatory cytokine IL-1*β*, the TLR2/6 ligand and bacterial product fibroblast-stimulating lipopeptide- (fsl-) 1, the TLR5 ligand and bacterial product flagellin, and the TLR3 ligand and viral dsRNA analogue polyinosinic:polycytidylic acid (poly(I:C)) were chosen as they have been shown to promote the expression of proinflammatory and prolabour mediators in human gestational tissues [21, 33, 34].

## 2. Materials and Methods

### 2.1. Ethics Statement

This study was approved by the Research Ethics Committee of Mercy Hospital for Women. Written, informed consent was obtained from all participating women.

### 2.2. Tissue Collection

Myometrium and fetal membranes were collected for two separate studies: expression studies and cell culture studies. All tissues were obtained from women who delivered healthy, singleton infants. Exclusion criteria were BMI > 30, abnormal antenatal glucose tolerance test results, any underlying medical conditions (for example, diabetes mellitus, macrovascular complications, polycystic ovarian syndrome, and preeclampsia), multiple pregnancies, and presence of fetal chromosomal abnormalities. Tissues were processed in the laboratory within 15 min of delivery.

#### 2.2.1. Tissue Collection for Expression Studies

The full clinical characteristics of the patients used for the expression studies are described elsewhere [35].

Myometrium was obtained from women at term (37–41 weeks' gestation) undergoing (i) elective Caesarean section in the absence of labour and (ii) emergency Caesarean section during active labour (*n* = 8 patients per group). Indications for Caesarean section in the absence of labour were breech presentation and/or previous Caesarean section. Indications for Caesarean section in the presence of labour were placenta praevia, fetal distress, and failure to progress. Myometrial biopsies were obtained from the upper margin of the lower uterine segment incision during Caesarean section. No patients underwent induction or augmentation of labour. Tissue samples were snap frozen in liquid nitrogen and immediately stored at −80°C.

Fetal membranes were obtained from women (i) at term undergoing elective Caesarean section in the absence of labour and (ii) at term after spontaneous labour, spontaneous membrane rupture, and normal vaginal delivery (*n* = 9 patients per group). Indications for Caesarean section were breech presentation and/or previous Caesarean section. No patients underwent induction or augmentation of labour. Tissue samples were snap frozen in liquid nitrogen and immediately stored at −80°C.

Fetal membranes were also obtained from women at preterm birth for two separate studies on preterm labour and preterm chorioamnionitis. For the preterm labour study, fetal membranes (amnion and choriodecidua) were obtained from women (i) undergoing Caesarean section in the absence of labour with intact membranes and (ii) after spontaneous labour and normal vaginal delivery (*n* = 9 patients per group). For the chorioamnionitis study, only amnion was collected as the choriodecidual tissue was degraded. Amnion was collected from women (i) undergoing Caesarean section in the absence of labour and (ii) undergoing Caesarean section in the absence of labour with histologically confirmed acute chorioamnionitis (*n* = 8 patients per group). Indications for preterm delivery (in the absence of labour) were placenta praevia, placental abruption, antepartum haemorrhage, or rhesus isoimmunisation. All preterm placentas were subject to histopathological examination and fetal membranes were swabbed for microbiological culture studies. Chorioamnionitis was diagnosed pathologically according to standard criteria [36].

#### 2.2.2. Tissue Collection for Cell Culture Studies

For the cell culture studies, fresh amnion and myometrium were obtained from women who delivered healthy, singleton infants at term (37–40 weeks' gestation) undergoing elective Caesarean section in the absence of labour. Primary amnion and myometrial cells were isolated and cultured as previously described [37, 38].

### 2.3. DREAM siRNA Transfection in Primary Myometrial and Amnion Cells

Primary myometrial and amnion cells were transfected with siRNA. Myometrial and amnion cells at approximately 50% confluence were transfected using Lipofectamine 3000 according to manufacturer's guidelines (Life Technologies; Mulgrave, Victoria, Australia). DREAM siRNA (siDREAM) and negative control (siCONT) were obtained from Ambion (Thermo Fisher Scientific; Scoresby, VIC, Australia). Myometrial cells were transfected with 50 nM siDREAM or 50 nM siCONT in DMEM/F-12 for 48 h followed by treatment with or without 100 pg/ml IL-1*β*, 250 ng/ml fsl-1, 1 *μ*g/ml flagellin, or 5 *μ*g/ml poly(I:C) for 24 h. Amnion cells were transfected with 50 nM siDREAM or 50 nM siCONT in DMEM/F-12 for 48 h followed by treatment with or without 100 pg/ml IL-1*β* for 24 h. After the final incubation, cells and media were collected and stored at −80°C until assayed as detailed below. Cell viability was assessed by the 3-(4,5-dimethyl-2-thiazolyl)-2,5-diphenyl-2H-tetrazolium bromide (MTT) proliferation assay as we have previously described [39]. The data is presented as fold change in expression relative to the expression level in the IL-1*β*, flagellin-, and fsl-1- or poly(I:C)-stimulated siCONT-transfected cells, which was set at 1. Each experiment was performed on amnion and myometrium obtained from six patients.

### 2.4. NF-*κ*B RelA Luciferase Activity

Possible interactions between DREAM and NF-*κ*B were determined using a luciferase assay, as previously described [40]. Primary myometrial cells, prepared as above, at ~70% confluence were transfected with 0.75 ng NF-*κ*B RelA reporter construct (Qiagen) using FuGENE HD transfection reagent (Promega, Alexandria, NSW, Australia). After 6 h, cells were transfected with 50 nM siDREAM or siCONT (as detailed above) for 48 h. The medium was then replaced with DMEM/F12 containing 0.5% BSA with or without 100 pg/ml IL-1*β*, 250 ng/ml fsl-1, 1 *μ*g/ml flagellin, or 5 *μ*g/ml poly(I:C), and the cells were incubated at 37°C for an additional 24 h. Cells were harvested in lysis buffer and luminescence activity was measured using a luciferase reporter assay kit (Life Research, Scoresby, Victoria, Australia) and Renilla luciferase flash assay kit (Thermo Fisher Scientific, Scoresby, Victoria, Australia), as per the manufacturer's instructions. The ratio of firefly luciferase level to Renilla luciferase level was determined and results are expressed as a ratio of normalised luciferase activity. The experiments were performed on myometrium obtained from six patients.

### 2.5. RNA Extraction and qRT-PCR

RNA extraction and qRT-PCR were performed as previously described [40]. Total RNA was extracted from tissues and cells using TRIsure reagent, as per the manufacturer's instructions (Bioline, Alexandria, NSW, Australia). RNA concentration and purity were measured using a NanoDrop ND1000 Spectrophotometer. RNA quality was determined via the* A*_260_ :* A*_280_ ratio. RNA was converted to cDNA using the high-capacity cDNA reverse transcription kit (Thermo Fisher Scientific; Scoresby, Vic, Australia) according to the manufacturer's instructions. The RT-PCR was performed using the CFX384 Real-Time PCR detection system (Bio-Rad Laboratories; Gladesville, NSW, Australia) using 100 nM of predesigned and validated QuantiTect primers (primer sequences not available) (Qiagen; Chadstone Centre, Vic, Australia). Average gene Ct values were normalised to the average YWHAZ and succinate dehydrogenase (SDHA) Ct values of the same cDNA sample. Fold differences were determined using the comparative Ct method.

### 2.6. Enzyme Immunoassays

IL-6 and IL-8 release were assessed using the CytoSet™ sandwich ELISA, as instructed (Life Technologies). MCP-1 and ICAM-1 release were assessed by sandwich ELISA from R&D Systems (Minneapolis, MN, USA), as instructed. The interassay and intra-assay coefficients of variation for all assays were less than 10%.

### 2.7. Gelatin Zymography

MMP-9 activity was assessed by gelatin zymography on conditioned media collected from primary amnion cells, as previously described [34]. Briefly, proteolytic activity was visualised as clear zones of lysis on a blue background of undigested gelatin. Gels were scanned and inverted using the ChemiDoc XRS system (Bio-Rad Laboratories), and densitometry was performed using the Quantity One Image analysis software (Bio-Rad Laboratories).

### 2.8. Statistical Analysis

Statistical analysis was performed using GraphPad Prism (GraphPad Software, La Jolla, CA). For two sample comparisons, an unpaired Student's *t*-test was used to assess statistical significance between normally distributed data; otherwise, the nonparametric Mann–Whitney *U* was used. For all other comparisons, the homogeneity of data was assessed by Bartlett's test, and, when significant, data were logarithmically transformed before analysis by a repeated measures one-way ANOVA (with LSD post hoc testing to discriminate among the means). Statistical significance was ascribed to a *p* value ≤ 0.05. Data is expressed as mean ± SEM.

## 3. Results

### 3.1. Effect of Term and Preterm Labour and Infection on DREAM Expression in Human Myometrium and Fetal Membranes

**Figure 1:**
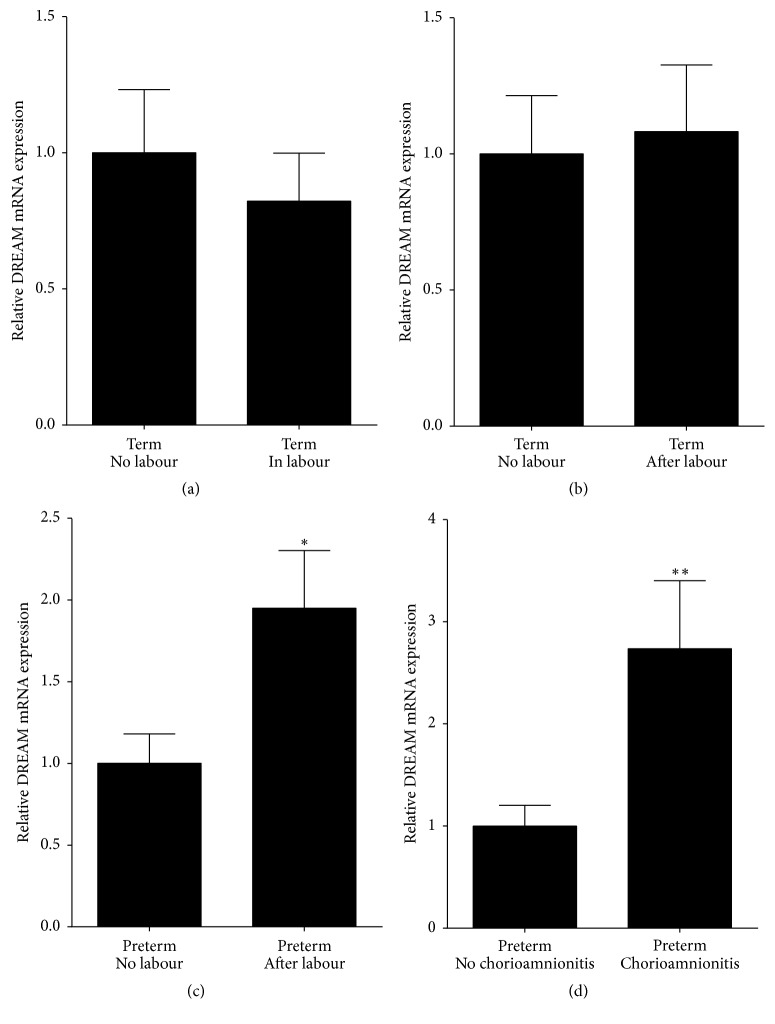
*DREAM expression in human myometrium and fetal membranes*. (a) Human myometrium was obtained from nonlabouring and labouring women at term Caesarean section (*n* = 8 patients per group). (b) Fetal membranes were obtained from women not in labour at term Caesarean section and women after term spontaneous labour onset and delivery (*n* = 9 patients per group). (c) Fetal membranes were obtained from women not in labour at preterm Caesarean section and women after preterm spontaneous labour onset and delivery (*n* = 9 patients per group). (d) Amnion was obtained from women at preterm Caesarean section with or without histological chorioamnionitis (*n* = 8 patients per group). DREAM mRNA expression was analysed by qRT-PCR. All data are displayed as mean ± SEM. ^*∗*^*p* ≤ 0.05 versus preterm no labour (Student's *t*-test); ^*∗∗*^*p* ≤ 0.05 versus preterm no chorioamnionitis (Student's *t*-test).

We first characterised the expression of DREAM in myometrium and fetal membranes from nonlabouring and labouring women. DREAM mRNA expression was not different in myometrium and fetal membranes obtained from labouring and nonlabouring women at term (Figures 1(a) and 1(b)). On the other hand, in fetal membranes obtained from women at preterm, DREAM mRNA expression was significantly higher in the labouring group compared to the nonlabouring group (Figure 1(c)). Furthermore, at preterm, DREAM mRNA expression was also significantly higher in amnion obtained from women with histologically confirmed chorioamnionitis compared to those without histologically confirmed chorioamnionitis (Figure 1(d)). Several attempts to quantify DREAM protein with commercially available antibodies were unsuccessful.

### 3.2. Effect of siDREAM on Proinflammatory Cytokines, Chemokines, and Adhesion Molecules in Primary Myometrial and Amnion Cells

Functional siRNA studies were performed to determine the role of DREAM in the regulation of prolabour mediators. For these studies, we used primary cells isolated from human myometrium or amnion and treated them with the proinflammatory cytokine IL-1*β*, the bacterial products fsl-1 or flagellin, and the viral dsRNA analogue poly(I:C) to induce inflammation associated with preterm labour. Following siRNA transfection, primary myometrial cells were treated with IL-1*β*, fsl-1, flagellin, and poly(I:C). Amnion cells were treated with IL-1*β* only. Efficacy of transfection was assessed by qRT-PCR. As compared to siCONT-transfected myometrial and amnion cells, siDREAM transfection resulted in a decrease in DREAM mRNA expression by approximately 75%. A MTT cell viability assay showed no difference in absorbance between siCONT- and siDREAM-transfected myometrial (0.63 ± 0.34 versus 0.66 ± 0.27) and amnion (1.49 ± 0.15 versus 1.45 ± 0.15) cells.

**Figure 2:**
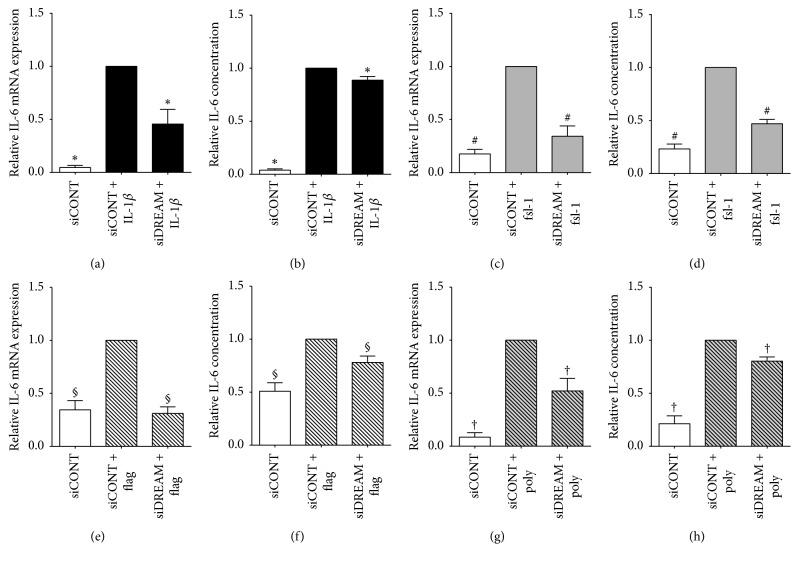
*Effect of siDREAM on the proinflammatory cytokine IL-6 in primary myometrial cells*. Primary myometrial cells were transfected with 50 nM siCONT or 50 nM siDREAM for 48 h and then treated with (a, b) 100 pg/mL IL-1*β*, (c, d) 250 ng/ml fsl-1, (e, f) 1 *μ*g/ml flagellin, or (g, h) 5 *μ*g/ml poly(I:C) for an additional 24 h (patients). (a, c, e, g) IL-6 mRNA expression was analysed by qRT-PCR. (b, d, f, h) The incubation media was assayed for concentration of IL-6 by ELISA. For all data, fold change was calculated relative to IL-1*β*-, fsl-1, and flagellin- or poly(I:C)-stimulated siCONT-transfected cells. Data are displayed as mean ± SEM. ^*∗*^*p* ≤ 0.05 versus IL-1*β*-stimulated siCONT-transfected cells; ^#^*p* ≤ 0.05 versus fsl-1-stimulated siCONT-transfected cells; ^§^*p* ≤ 0.05 versus flagellin-stimulated siCONT-transfected cells; ^†^*p* ≤ 0.05 versus poly(I:C)-stimulated siCONT-transfected cells (one-way ANOVA).

**Figure 3:**
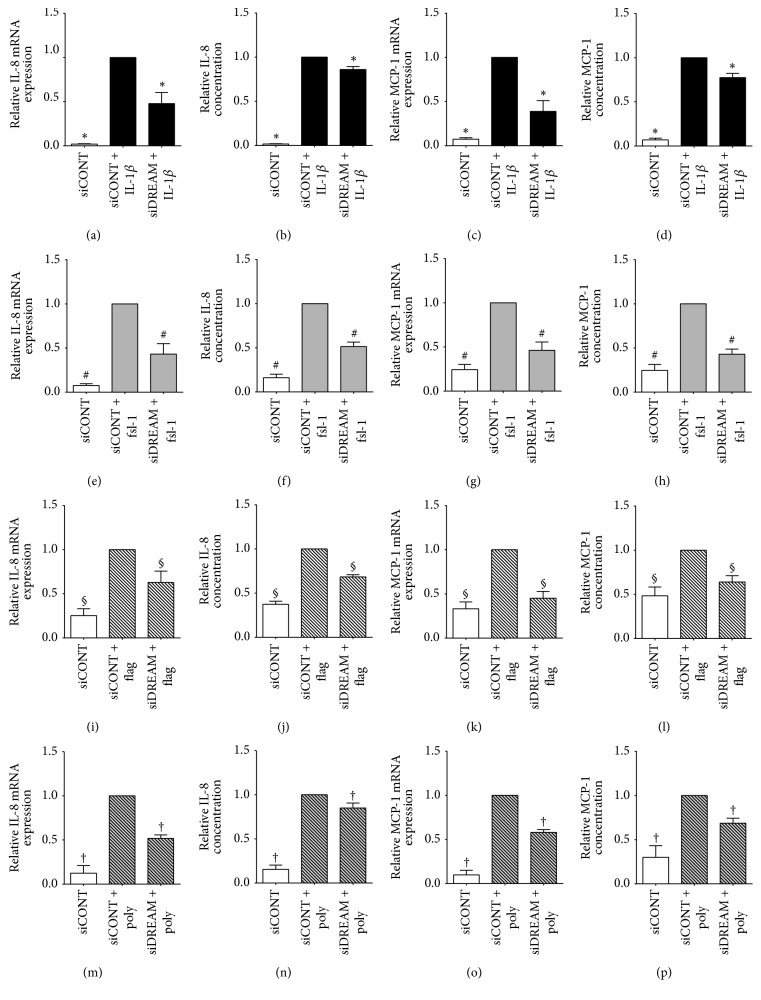
*Effect of siDREAM on chemokines in primary myometrial cells*. Primary myometrial cells were transfected with 50 nM siCONT or 50 nM siDREAM for 48 h and then treated with (a–d) 100 pg/mL IL-1*β*, (e–h) 250 ng/ml fsl-1, (i–l) 1 *μ*g/ml flagellin, or (m–p) 5 *μ*g/ml poly(I:C) for an additional 24 h (*n* = 6 patients). (a, c, e, g, i, k, m, o) IL-8 and MCP-1 mRNA expression were analysed by qRT-PCR. (b, d, f, h, j, l, n, p) The incubation media was assayed for concentration of IL-8 and MCP-1 by ELISA. For all data, fold change was calculated relative to IL-1*β*-, fsl-1, and flagellin- or poly(I:C)-stimulated siCONT-transfected cells. Data are displayed as mean ± SEM. ^*∗*^*p* ≤ 0.05 versus IL-1*β*-stimulated siCONT-transfected cells; ^#^*p* ≤ 0.05 versus fsl-1-stimulated siCONT-transfected cells; ^§^*p* ≤ 0.05 versus flagellin-stimulated siCONT-transfected cells; ^†^*p* ≤ 0.05 versus poly(I:C)-stimulated siCONT-transfected cells (one-way ANOVA).

**Figure 4:**
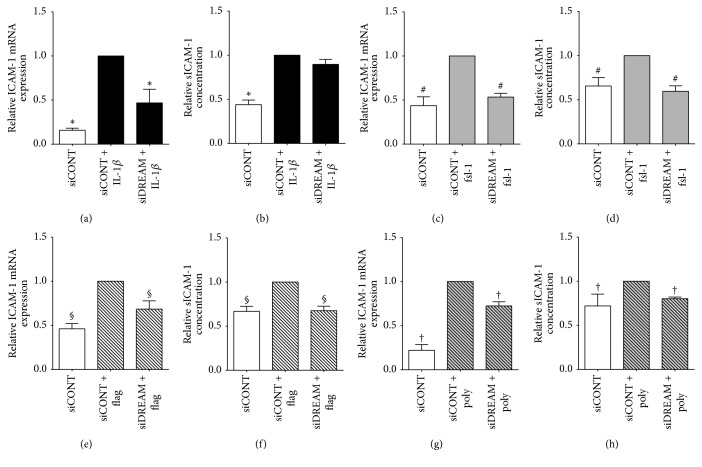
*Effect of siDREAM on the adhesion molecule ICAM-1 in primary myometrial cells*. Primary myometrial cells were transfected with 50 nM siCONT or 50 nM siDREAM for 48 h and then treated with (a, b) 100 pg/mL IL-1*β*, (c, d) 250 ng/ml fsl-1, (e, f) 1 *μ*g/ml flagellin, or (g, h) 5 *μ*g/ml poly(I:C) for an additional 24 h (*n* = 6 patients). (a, c, e, g) ICAM-1 mRNA expression was analysed by qRT-PCR. (b, d, f, h) The incubation media was assayed for concentration of ICAM-1 by ELISA. For all data, fold change was calculated relative to IL-1*β*-, fsl-1, and flagellin- or poly(I:C)-stimulated siCONT-transfected cells. Data are displayed as mean ± SEM. ^*∗*^*p* ≤ 0.05 versus IL-1*β*-stimulated siCONT-transfected cells; ^#^*p* ≤ 0.05 versus fsl-1-stimulated siCONT-transfected cells; ^§^*p* ≤ 0.05 versus flagellin-stimulated siCONT-transfected cells; ^†^*p* ≤ 0.05 versus poly(I:C)-stimulated siCONT-transfected cells (one-way ANOVA).

**Figure 5:**
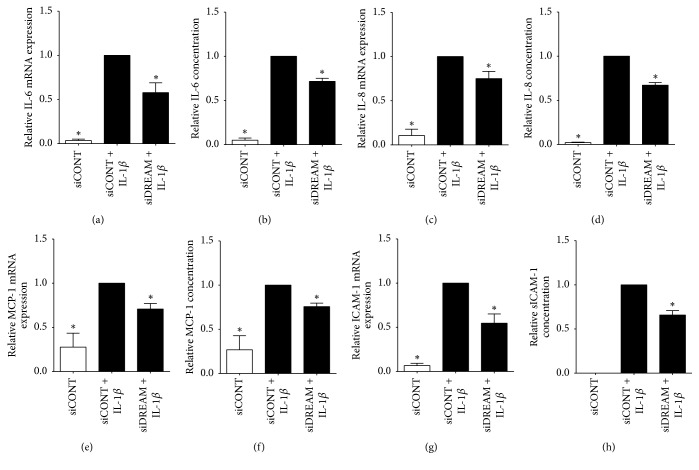
*Effect of siDREAM on proinflammatory cytokines, chemokines, and adhesion molecules in primary amnion cells*. Primary amnion cells were transfected with 50 nM siCONT or 50 nM siDREAM for 48 h and then treated with 100 pg/mL IL-1*β* for an additional 24 h (*n* = 6 patients). (a, c, e, g) IL-6, IL-8, MCP-1, and ICAM-1 mRNA expression were analysed by qRT-PCR. (b, d, f, h) The incubation media was assayed for concentration of IL-6, IL-8, MCP-1, and sICAM-1 by ELISA. For all data, fold change was calculated relative to IL-1*β*-stimulated siCONT-transfected cells. Data are displayed as mean ± SEM. ^*∗*^*p* ≤ 0.05 versus IL-1*β*-stimulated siCONT-transfected cells (one-way ANOVA).

The effect of siDREAM on proinflammatory cytokines, chemokines, and adhesion molecules is depicted in Figures 2–4 for myometrial cells and Figure 5 for amnion cells. In siCONT-transfected myometrial cells, treatment with IL-1*β*, fsl-1, flagellin, and poly(I:C) significantly increased expression of IL-6 (Figure 2), IL-8, and MCP-1 (Figure 3). In siDREAM transfected cells, there was a significant attenuation of IL-6, IL-8, and MCP-1 mRNA expression and secretion when stimulated with all treatments. There was also a significant decrease in fsl-1, flagellin, and poly(I:C)-induced ICAM-1 mRNA expression and secretion in siDREAM transfected cells (Figure 4). There was a significant decrease in IL-1*β*-induced ICAM-1 mRNA expression, but no effect on secretion of sICAM-1 in siDREAM transfected cells (Figure 4). Similar results were obtained in amnion cells, where siDREAM transfected cells displayed an attenuation of IL-1*β*-induced IL-6, IL-8, MCP-1, and ICAM-1 mRNA expression and secretion (Figure 5).

### 3.3. Effect of siDREAM on MMP-9 in Primary Amnion Cells

**Figure 6:**
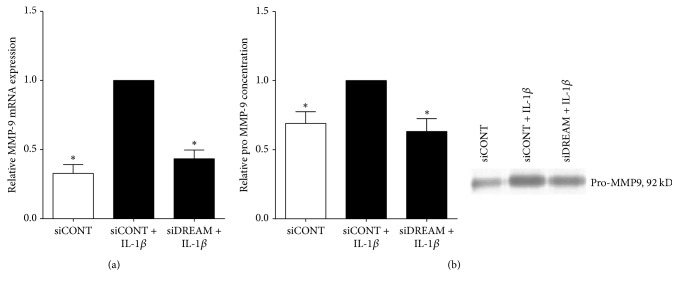
*Effect of siDREAM on the ECM degrading enzyme MMP-9 in primary amnion cells*. Primary amnion cells were transfected with 50 nM siCONT or siDREAM for 48 h and then treated with 100 pg/mL IL-1*β* for an additional 24 h (*n* = 6 patients). (a) MMP-9 mRNA expression was analysed by qRT-PCR. (b) The incubation media was assessed for pro-MMP-9 proteolytic activity using gelatin zymography. Representative zymogram from one patient is shown. For all data, fold change was calculated relative to IL-1*β*-stimulated siCONT-transfected cells. Data are displayed as mean ± SEM. ^*∗*^*p* ≤ 0.05 versus IL-1*β*-stimulated siCONT-transfected cells (one-way ANOVA).

We also assessed the effect of siDREAM on the expression of the ECM degrading enzyme MMP-9 in primary amnion cells. As expected, IL-1*β* increased MMP-9 mRNA expression and secretory pro-MMP-9 levels in siCONT-transfected amnion cells (Figure 6). The effect of siDREAM was a significant suppression of IL-1*β*-induced MMP-9 mRNA expression and pro-MMP-9 production.

### 3.4. Effect of siDREAM on NF-*κ*B RelA Transcriptional Activity

**Figure 7:**
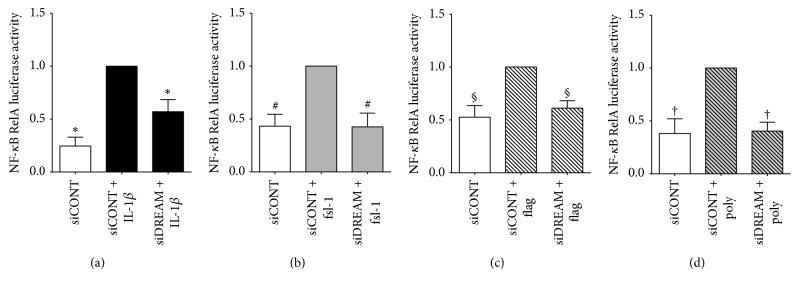
*Effect of siDREAM on NF-κB RelA transcriptional activity*. Primary myometrial cells were transfected with 300 ng/ml NF-*κ*B RelA reporter construct for 6 h, transfected with 50 nM siCONT or siDREAM for 48 h, and then treated with (a) 100 pg/ml IL-1*β*, (b) 250 ng/ml fsl-1, (c) 1 *μ*g/ml flagellin, or (d) 5 *μ*g/ml poly(I:C) for an additional 24 h (*n* = 5-6 patients). Promoter activity was normalised to* Renilla *expression. Fold change was calculated relative to IL-1*β*-, fsl-1, and flagellin- or poly(I:C)-stimulated siCONT-transfected cells. Data are displayed as mean ± SEM. ^*∗*^*p* ≤ 0.05 versus IL-1*β*-stimulated siCONT-transfected cells; ^#^*p* ≤ 0.05 versus fsl-1-stimulated siCONT-transfected cells; ^§^*p* ≤ 0.05 versus flagellin-stimulated siCONT-transfected cells; ^†^*p* ≤ 0.05 versus poly(I:C)-stimulated siCONT-transfected cells (one-way ANOVA).

Finally, we determined if the effects of siDREAM on prolabour mediators may be elicited through NF-*κ*B; Figure 7 demonstrates the effect of siDREAM on NF-*κ*B RelA transcriptional activity. In siCONT-transfected cells, NF-*κ*B RelA transcriptional activity was significantly augmented by IL-1*β*, fsl-1, flagellin, and poly(I:C) treatment. A significant reduction in IL-1*β*-, fsl-1-, and flagellin- and poly(I:C)-induced NF-*κ*B RelA transcriptional activity was observed in siDREAM transfected cells.

## 4. Discussion

A greater understanding of the mechanisms of human parturition is necessary to design new and effective strategies for the prevention of preterm labour. Here, DREAM is identified as a novel therapeutic target. This study is the first to investigate expression and function of DREAM in human myometrium and fetal membranes. While DREAM expression is not altered by labour at term, its expression is significantly increased in fetal membranes after preterm labour and in amnion with histological preterm chorioamnionitis when compared to amnion without histological preterm chorioamnionitis. Functional studies in primary myometrial and amnion cells revealed that DREAM is involved in the production of proinflammatory and prolabour mediators induced by IL-1*β*, fsl-1, flagellin, and poly(I:C). Additionally, NF-*κ*B RelA transcriptional activity was significantly reduced in siDREAM transfected myometrial cells, suggesting that DREAM may regulate prolabour mediators via NF-*κ*B signalling.

Increased DREAM expression is implicated in both physiological and pathological inflammatory states, including pain sensation (a hallmark of inflammation), Alzheimer's disease, and preeclampsia [27, 29, 32]. Inflammation is a common feature of human labour, with increased production of proinflammatory cytokines (such as IL-1*β*) by leukocytes infiltrating the myometrium, cervix, and fetal membranes [6, 7]. In this study, DREAM expression was similar between myometrium and fetal membranes obtained from nonlabouring and labouring women at term. On the other hand, at preterm, DREAM expression was significantly increased in fetal membranes from labouring women compared to nonlabouring women. This suggests that DREAM is not involved in the processes of healthy labour at term but is involved in the pathological activation of labour at preterm. Preterm labour is associated with increased inflammation in myometrium, fetal membranes, and amniotic fluid in the absence of infection, but also in an exaggerated manner in cases of infection [13]. We found that DREAM mRNA expression was significantly increased in preterm amnion with histological chorioamnionitis compared to amnion without histological chorioamnionitis. The fact that DREAM mRNA expression was increased in amnion with chorioamnionitis suggests that this increase may be caused by infection. Further studies are required to determine the role of proinflammatory and infectious stimuli in regulating DREAM expression. While it would be of great benefit to determine the expression of DREAM in myometrium from preterm deliveries with or without infection, obtaining such samples is extremely difficult. Notably, we only assessed DREAM mRNA expression; protein data are needed to verify these findings. Notwithstanding these limitations, the data collectively suggest that DREAM upregulation is more prevalent in states of heightened inflammation. Functional studies were then performed to determine whether DREAM regulates proinflammatory and prolabour mediators. For these studies, the proinflammatory cytokine IL-1*β*, two bacterial products (fsl-1 and flagellin), and one viral product (poly(I:C)) were used to mimic inflammation associated with preterm labour [33, 41].

IL-1*β* is a proinflammatory cytokine released from infiltrating leukocytes in intrauterine tissues [7] that are central to the terminal pathways of human labour and delivery. Elevated IL-1*β* expression is found in the human myometrium, amnion, amniotic fluid, and cervix in association with term and preterm labour [6, 7, 42], while in intra-amniotic administration of IL-1*β* it induces preterm delivery in mice and rhesus monkeys [43, 44]. In intrauterine tissues, IL-1*β* has been shown to induce expression of chemokines, adhesion molecules, MMPs, and contractions associated proteins [10, 45–48]. In this study, siDREAM knockdown in primary myometrial and amnion cells was associated with significant decrease in IL-1*β*-induced expression and secretion of the proinflammatory cytokine IL-6, the chemokines IL-8 and MCP-1, and the adhesion molecule ICAM-1. Collectively, however, the results of this study indicate that DREAM is involved in the genesis of proinflammatory and prolabour mediators induced by IL-1*β*.

Activation of TLRs within intrauterine tissues is an important catalyst of preterm labour, with animal models identifying a role for TLR2 and TLR3 in particular. TLR2 ligation by bacterial products can induce preterm birth in mice, while, conversely, mice lacking TLR2 demonstrate reduced expression of inflammatory and contractile genes as well as delayed timing of labour [49, 50]. There is also a synergy between TLR2 and TLR3, as combined stimulation using both agonists leads to induction of the inflammatory response and preterm labour in the mouse, caused by the alternate ligand [51]. In mice, advancing gestation is correlated with enhanced amniotic fluid expression of TLR2 and in humans TLR2 expression is increased in myometrium and fetal membranes in association with spontaneous term and preterm labour with evidence of chorioamnionitis [50, 52, 53]. Additionally, fsl-1, flagellin, and poly(I:C) (TLR2, TLR5, and TLR3 agonists, respectively) upregulate the expression and release of proinflammatory and prolabour mediators in myometrium and fetal membranes [21, 33, 41, 54]. DREAM has been shown to play an important role in regulating inflammation in response to infection. In a model of polymicrobial sepsis in DREAM-deficient mice, decreased IL-6, MCP-1, and ICAM-1 release was found in bronchoalveolar lavage fluid [24]. In support of these findings, our study demonstrates that siDREAM knockdown in primary myometrial cells associated with a significant decrease in fsl-1-, flagellin-, and poly(I:C)-induced expression and secretion of the proinflammatory cytokines IL-6, the chemokines IL-8 and MCP-1, and the adhesion molecule ICAM-1. This suggests that DREAM plays an important role in TLR signalling pathways associated with preterm labour.

Recent studies have identified DREAM as a regulator of NF-*κ*B, a proinflammatory transcription factor critical to the synthesis of prolabour mediators [15, 16]. NF-*κ*B signalling components, including RelA, have been identified in numerous gestational cells and tissues, with labour-associated increases in NF-*κ*B signalling activity reported in myometrium, cervix, and amnion [55–58]. Decreased expression of multiple NF-*κ*B signalling components has been demonstrated in lung vascular endothelial cells and neutrophils from DREAM-deficient mice [24, 25]. In our study, siDREAM knockdown was associated with a significant decrease in IL-1*β*-, fsl-1-, flagellin-, and poly(I:C)-induced NF-*κ*B RelA transcriptional activity. Altogether, these findings suggest that, in human myometrium, DREAM is a mediator of the NF-*κ*B signalling pathway, corroborating the potential of DREAM as a therapeutic target for the prevention of preterm labour.

Despite clinical interventions and extensive research, preterm birth rates continue to rise [59]. This may be due to an incomplete understanding of the mechanisms of human labour. This study suggests a role for DREAM in inflammation- and/or infection-induced preterm birth. DREAM mRNA expression is increased with preterm labour and in preterm amnion with histological chorioamnionitis, with loss-of-function studies suggesting that DREAM may regulate proinflammatory and prolabour mediators via NF-*κ*B signalling in human myometrium and fetal membranes. Thus, inhibition of DREAM represents a novel therapeutic strategy for the prevention and management of preterm labour. Further studies are required to fully ascertain the role of DREAM in the processes of parturition.
